# Effect of Maxillary Expansion on the Maxillary Arch Width in Patients with Bilateral Cleft Palate: A Review

**DOI:** 10.3390/children10050762

**Published:** 2023-04-23

**Authors:** Omar H. Alkadhi, Lamis Hejab Alotaibi, Rowaida R. Alrashoud, Mohammed Hamad Almutairi, Huda Ali Al Matar, Sreekanth Kumar Mallineni

**Affiliations:** 1 Preventive Dentistry Department, College of Dentistry, Riyadh Elm University (REU), Riyadh 13244, Saudi Arabia; 2 Department of Preventive Dental Science, College of Dentistry, Majmaah University, Al Majmaah 11952, Saudi Arabia; 3 College of Dentistry, Riyadh Elm University (REU), Riyadh 13244, Saudi Arabia; 4 General Dentist, College of Dentistry, Prince Sattam bin Abdulaziz University, Al-Kharj 16278, Saudi Arabia; 5 General Dentist, Ministry of Health, Dammam 32242, Saudi Arabia; 6 Pediatric Dentistry, Dr. Sulaiman Al Habib Hospital, Ar Rayyan, Riyadh 14212, Saudi Arabia; 7 Center for Transdisciplinary Research (CFTR), Saveetha Dental College, Saveetha Institute of Medical and Technical Sciences, Saveetha University, Chennai 600077, India; 8 Division for Globalization Initiative, Liaison Center for Innovative Dentistry Graduate School of Dentistry, Tohoku University, Sendai 980-8575, Japan

**Keywords:** slow maxillary expansion, rapid maxillary expansion, maxillary arch width, cleft palate

## Abstract

Objectives: To perform a comprehensive review of the literature to compare the effects of slow maxillary expansion (SME) and rapid maxillary expansion (RME) on maxillary arch width in patients with bilateral cleft palate. Methods: The databases include Medline, PubMed, Cochrane (CENTRAL) and (CDSR), OpenGrey, and ClinicalTrials.gov were searched for relevant studies that met the eligibility criteria published before or on 31 October 2022. The search was confined to the English language. The selection of eligible studies and collection of data were performed independently. Risk of bias assessment was conducted using the Cochrane Risk of Bias tool 2.0. Results: Two randomized controlled trials were available based on the search in the published literature. Both studies compared arch width between SME and RME in cleft palate patients and digitals casts and three-dimensional images used for the evaluation. A moderate risk of bias was evident in the available studies. Conclusions: Both SME and RME can achieve similar amounts of maxillary expansion in patients with bilateral cleft palate.

## 1. Introduction

The most common types of craniofacial abnormalities are the cleft lip and cleft palate [1]. The incidence of this abnormality was reported to be one in seven hundred live births [2]. The genesis of this craniofacial deformity is thought to be multifaceted, and there is a possibility that factors such as geography, race, ethnicity, and socioeconomic status may all play a role in its prevalence [3,4]. Having a cleft lip and/or palate (CL/P) on one side of the mouth is more common than having it on both sides, and males are more likely to be affected by this deformity than females [5]. In most cases, it is accompanied by growth deficits in the anterior-posterior, lateral, transverse, and vertical directions [6,7]. The therapy for cleft palate requires teams of specialists from a variety of fields as well as many treatment phases [8]. Following maxillary surgery, the patient’s capacity for transverse maxillary growth may be limited. Individuals who have a cleft lip and/or palate have an increased risk of developing dental plaque, which can put them at an increased risk for a variety of dental illnesses affecting both the soft tissue and the hard tissue [9,10]. In comparison to the population as a whole, the likelihood of these individuals having malocclusions is significantly increased. Previous research has classified the presence of class III malocclusions in patients with cleft lip and/or palate, and they reported a prevalence of 62% in these patients [11,12,13]. Some of these patients were born with an incomplete or incompletely formed palate. In total, 8.6% of patients with oral clefts were found to have anterior or posterior crossbites, according to a study [11] conducted in Japan. In addition, a study conducted in Korea [12] indicated that the frequency of malocclusions in trans-foramen incisor clefts was 76.3%. In comparison, the frequency of pre-foramen incisor clefts was 42.1%. Such dental abnormalities in children with clefts and maxillary hypoplasia lead to a variety of modifications in the vertical dimensions of the occlusions [8,9,10,11,12]. These include reverse overjet, class III malocclusion, anterior crossbite, and posterior crossbite.

According to the findings of a study [13] conducted in Brazil, patients with different types of oral clefts have varying requirements for orthodontic treatment. The authors also mentioned that in order for patients to be adequately treated, they need to have specialized dental and oral health care. In patients who have a cleft palate, it is not totally obvious which therapy regimen offers the greatest results regarding when it comes to maxillary expansion. As part of the therapy for maxillary constriction [14], it is usual practice to carry out maxillary expansion in the goal of expanding the maxillary arch in order to provide more space for the upper teeth. When working to treat children who have cleft palate, the maxillary expansion process may involve either a rapid or a delayed palatal expansion using orthodontics. This is dependent on the severity of the condition being treated. Patients diagnosed with cleft palate are often subjected to either a slow or quick maxillary expansion prior to the performance of a secondary bone graft [15]. These are the two primary expansion techniques [16,17]. A quad-helix expander is utilized when performing a slow maxillary expansion, but a Haas or Hyrax expander is utilized when performing a rapid maxillary expansion (RME). A maxillary expansion treatment that is universally acknowledged as the standard of care is not yet available for patients with cleft palates. Although some orthodontists favor the rapid maxillary expansion (RME) technique, others opted for the slow maxillary expansion (SME) [8,17].

In growing patients who do not have oral clefts, a procedure called rapid maxillary expansion (RME) can correct maxillary constriction and posterior crossbites. This is accomplished by RME, which opens the midpalatal suture. The RME effect results in transverse bone gains, which, in turn, increase the maxillary dental arch perimeter and the buccal inclination of the maxillary first permanent molars, while also causing slight changes to the buccal bone. In addition, the RME effect may cause some slight shifts in the position of the maxillary first permanent molars. On the other hand, it has been suggested that slow maxillary expansion (SME) shows essentially dentoalveolar effects, with smaller orthopedic repercussions. This is something that has been discovered through research. This is something that can be discovered in the research that has been made public. There was a greater bodily displacement of maxillary first permanent molars in the maxillary base when compared to RME, and there was a greater buccal bone loss when compared to rapid maxillary expansion. Both of these differences were observed in the maxillary base. However, given that patients with BCLP do not have midpalatal sutures, it is possible that the dentoskeletal effects of slow and rapid maxillary expansion will have different outcomes for these patients. This is because slow and rapid maxillary expansion are both mentioned above. Maxillary expansions are a common procedure that patients undergo prior to secondary alveolar bone grafting. This is done in order to make more room in the maxilla. There is still a lack of substantial evidence to suggest which treatment modality is superior for maxillary expansion in non-cleft patients who have posterior crossbites [18,19,20]. It was important to find out how SME and RME affected patients with both cleft lips and palates. Therefore, the aim of the paper was to conduct a review to compare the impact of SME and RME on maxillary width in patients with bilateral cleft lip and palate.

## 2. Methods

### 2.1. Registration and Protocol

This study was registered at the research center at Riyadh Elm University with registration number: SRS/2020/21/200 and IRB approval number: SRS/2020/21/200/195.

### 2.2. Eligibility Criteria

Inclusion criteria include the following:

- Study Design: randomized controlled clinical trials;
- Population: human participants in mixed dentition with bilateral cleft palate;
- Intervention: slow and rapid maxillary expansion;
- Outcome Measures: maxillary width;
- Only English language;
- Articles published before 31 October 2022.

Exclusion criteria include the following:

- Human studies with no comparison group;
- Studies with outcomes not related to dental alveolar changes;
- Unilateral cleft palate patients;
- Only rapid maxillary expansion;
- Only slow maxillary expansion;
- Studies involving patients other than mixed dentition;
- Other than randomized controlled trials and case reports.

Participants: bilateral cleft lip and palate adults and children; 

Study designs: randomized control trials;

Interventions studies that had used SME and RME as the interventions; 

Outcomes: maxillary arch expansion;

Primary search: published on or before 31 October 2022.

### 2.3. Information Sources and Search Strategy

The keywords including “cleft”, “lip”, “palate”, “bilateral”, “expansion”, and “Maxillary width” were used in various combinations for search in the databases. An electronic search was conducted in Medline via PubMed, Cochrane (CENTRAL) and (CDSR), OpenGrey, and ClinicalTrials.gov. Reference lists of the included articles were manually searched for relevant publications from the earliest available records up until 31 October 2022.

### 2.4. Selection Process

Titles and abstracts were screened, duplicates were removed, and inclusion and exclusion criteria were applied by three authors (H.A., L.A., and R.A.). Disagreements were resolved by a fourth author (O.A.).

### 2.5. Data Collection Process, Data Items, and Effect Measures

A specific data extraction form was used to extract data. Data included in the state were: year of publication, origin, sample size, interventions/comparators, outcomes, assessment methods, timeframe, and mean changes in maxillary width after expansion. The included studies were analyzed, and data were retrieved from each one using the preset list of outcomes of interest. This was carried out by a total of two reviewers, who were also responsible for designing and testing the data extraction form in advance of its actual implementation. Two of the reviewers were responsible for collecting the data from each of the studies, but they might consult the third reviewer if they were in disagreement.

## 3. Results

### Study Selection and Characteristics

**Figure 1:**
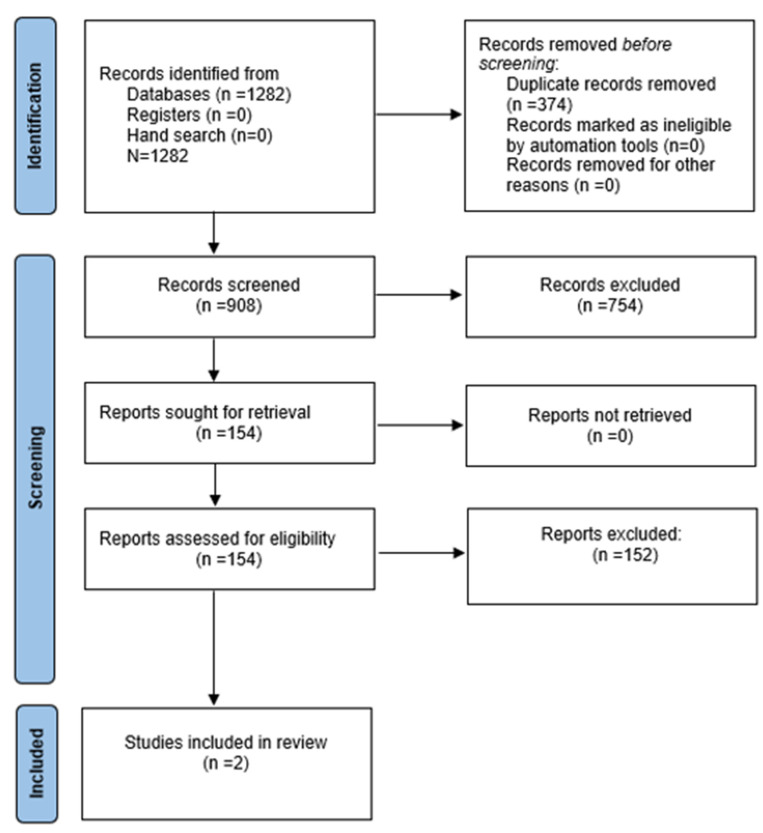
PRISMA flowchart used in the study.

The aforementioned keyword combinations were used to search through the various databases. The search yielded a total of 1282 titles. The manual search that was conducted in gray literature did not identify any additional citations that were relevant to the search question. In total, 908 articles remained after the duplicates were taken from the list. Following this, an initial screening was carried out, after which only 154 articles were selected to go through the process of having eligibility criteria. Just 2 articles among them compared the maxillary arch width of patients with bilateral cleft lip and palate who had been treated with SME and RME. Only 2 studies were taken into consideration for inclusion in the qualitative analysis. Figure 1 presents the PRISMA flowchart.

The review includes a total of two randomized controlled studies and the details of the included studies are shown in Table 1. These published studies were reported from Brazil. Both studies [21,22] analyzed 96 patients with bilateral cleft palate to determine the difference in arch width between SME and RME. In one study [21], 50 patients with bilateral cleft lip and palate participated, and in another study [22], the researchers reported on 46 patients. Table 2 provides a summary of the traits and criteria that were considered. There were a total of 96 participants in the study; 71 were males and 25 were females. De Medeiros Alves et al. [21] used digital casts for evaluation, while de Almeida et al. [22] used three-dimensional images for elevation. In their respective investigations, the SME and RME periods were each given values of 11 ± 4.58 months and 7.2 ± 3.51 months, respectively. De Medeiros Alves et al. [21] assessed measures in the canine, first premolar, second premolar, and molar regions, whereas de Almeida et al. [22] evaluated the alterations in the premolar region and the molar region. In both investigations, patients who were treated with SME had somewhat longer maxillary arches than patients who were treated with RME. This difference was statistically significant. Although there was no evidence of any major side effects, several patients who underwent rapid maxillary expansion reported feeling pressure in the area behind their teeth, beneath their eyes, and in the region surrounding their nose after receiving treatment. However, these symptoms dissipated very instantly and did not cause any significant discomfort [21,22]. De Almeida et al. [22] reported that patients with complete bilateral cleft lip and palate, slow and rapid maxillary expansions resulted in equivalent orthopedic, dental, and alveolar bone plate changes. In the mixed dentition, both appliances showed considerable skeletal transverse gains with negligible periodontal bone alterations; however, treatment time for RME was shorter than that reported for SME. However, de Medeiros Alves et al. [21] reported that the maxillary dental arch changes in patients with complete bilateral cleft lip and palate appear to be similar whether the expansion is slow or rapid. The treatment time for slow maxillary expansion is longer than that for RME.

## 4. Discussion

The transverse maxillary deficit, also known as posterior crossbite, occurs when the mandibular teeth come into buccal contact with the maxillary teeth [1]. Both the skeleton and the teeth could be to blame for posterior crossbites [12]. It does not matter what kind it is; posterior crossbite does not correct itself and needs to be treated once it has been identified. This will allow for optimal coordination of the maxillary and mandibular dental arches, prevent functional shifts and wear on the permanent teeth, and protect against dentofacial asymmetry and temporomandibular joint disorder [13,14]. As a result, maxillary expansion has become very popular in the orthodontic community and is now a common part of many orthodontic treatments [15]. Although it is recommended to correct transverse defects relatively early, up to the skeletal development spurt [9], the midpalatal suture fusion is inadequately connected with patient age and gender [10]. As a result, clear-cut indications for surgically aided maxillary expansion can be indeterminate. The effects of maxillary enlargement went beyond the maxilla and spread to most of the structures that were nearby [11,12]. Not only are orthodontist able to increase the transverse palatal dimension, but they are also able to influence the sagittal and vertical facial proportions [13], the mandible with its temporomandibular articulatory system [14], the airway spaces [15], and more. This is made possible by the intimate articulation between the maxilla and the mandible. Additionally, the patient age range that can be successfully treated using the basic maxillary expansion protocols has expanded thanks to the development of bone-anchored maxillary expansion [16] and the recent modalities for detecting midpalatal suture maturation [10]. 

Patients with BCLP who have undergone lip and palate repair at a young age typically have severe deficiencies in maxillary growth, as evidenced by maxillary dental arch constrictions and posterior cross bites. Patients with BCLP who did not undergo lip and palate repair at a young age typically have normal maxillary growth. Patients diagnosed with BCLP who underwent lip and palate reconstruction at an older age often do not display these symptoms. In patients who have been diagnosed with BCLP, it is usual for the maxillary expansion component of their orthodontic treatment to be required. When it comes to expanding the maxilla in preparation for getting secondary alveolar bone grafting, there is no one specific approach that should be followed. Some rehabilitation facilities recommend using slow maxillary expansion (SME) with the quad-helix appliance and its different iterations in order to address maxillary dental arch constriction. This is done in order to rectify the narrowing of the maxillary dental arch. On the other side, there are those who decide to go with rapid maxillary expansion, also known as RME. This procedure can be done with expanders of the Haas or Hyrax type. SME continues to encourage a mainly posterior tooth inclination in patients who do not have oral clefts, despite the fact that prior research indicated some bone development in cranio-maxillary sutures when oral clefts are not present. The opening of the midpalatal suture and dental movement in patients who do not have cleft palates is evidence that RME causes a bigger magnitude of forces to be released and promotes skeletal consequences. This can be seen in patients who have undergone RME treatment.

Studies that compare SME to RME in non-cleft patients may be found in abundance in the orthodontic literature [23]. When patients began their orthodontic treatment before the age of six, they demonstrated a better response in anterior maxillary expansion [24], which resulted in a significant improvement in the dental arch relationship. The clinical findings imply that maxillary expansion using the Quad Helix appliance is an acceptable alternative to conventional rapid maxillary expansion appliances among cleft patients [25,26,27,28,29]. This conclusion is based on the fact that the appliance is able to expand the maxilla more effectively. The treatment of clefts presents a difficulty that spans multiple disciplines for the teams who are involved in treatment. When treating individuals who have clefts, orthodontists typically utilize expansion in order to overcome maxillary constriction. There is an ongoing debate in orthodontic literature about whether or not SME and RME should be used [16,17,18]. As a result, this review was carried out to examine the effects of SME and RME on changes in the breadth of the maxillary arch in individuals who were born with both types of cleft palate. Within the scope of the review were randomized controlled studies. Both studies were found to have a risk of bias that was considered to be moderate. On the other hand, this danger could not be avoided because the intervention could not be concealed from either the participants or the workers.

SME and RME were found to be helpful in expanding the maxillary arch width in patients with bilateral clefts. When comparing the two methods in terms of maxillary arch width after expansion, no discernible difference was found between them. When using RME, expansion can be accomplished in a matter of weeks; however, when using SME, the same growth can take several months to accomplish. As a result, it may be prudent to advocate the use of RME rather than SME in individuals who have cleft palate on both sides [21,22]. In spite of the fact that there were worries over the adverse effects of RME in comparison to SME, such as lower buccal and lingual bone thickness, it was discovered that these adverse effects were not statistically different between the two methods of expansion [22]. In contrast, the RME and SME groups of patients with unilateral cleft lip and palate had similar dentoalveolar results in research that was conducted across multiple centers. In addition, the authors saw a more significant expansion in the RME group in comparison to the SME group [26].

In one randomized controlled research, the results of rapid maxillary expansion (RME) using an appliance called a Hyrax and slow maxillary expansion (SME) using an appliance called a quad-helix were compared six months following the expansion of patients who had bilateral cleft lip and palate. The evaluation was carried out using computerized models. It was discovered that the arch width could be successfully expanded by using either SME or RME. On the other hand, it was discovered that RME was more effective in expanding both the length of the arch and the depth of the palatal region, but SME was not successful in achieving either of these goals [21]. Using three-dimensional pictures, researchers in Canada were able to see the same patterns of skeletal extension in healthy persons after six months of treatment with either bone-anchored or tooth-anchored RME [27]. Cone-beam computed tomography, or CBCT, was used by Garib et al. for a study on RME employing Hyrax (tooth-borne) and Haas-type (tooth tissue-borne), and the researchers found that both groups experienced identical orthopedic effects [28]. Cone-beam computed tomography was utilized in yet another randomized controlled trial with the purpose of comparing the effects of Haas/Hyrax RME with SME with quad-helix. After 4–6 months of active expansion, a comparison was undertaken, and it was revealed that both the SME and RME induced an increase in maxillary arch width [22]. This was reported after the active expansion phase had been completed. RME and SME might have similar effects on vertical and sagittal alterations in children with bilateral cleft lip and palate (BCLP), according to the findings of a secondary analysis carried out by the same research group [29].

Both SME and RME were shown to lower bone height and thickness in patients who did not have cleft palates, with the SME group seeing a greater loss [23]. Both SME and RME showed similar changes in alveolar bone height, level of attachment, and periodontal probing depth in comparison to a control (no expansion) group when it came to periodontal alterations [30]. This was seen when comparing SME to RME in terms of periodontal changes. In a separate piece of research [31], the effects of SME and RME were studied utilizing a variety of periodontal markers. Between the two treatment approaches, there was found to be no discernible change in either the plaque index or the bleeding index, as well as the probing depth. When compared to SME, reports from patients without cleft palate suggested that RME was more likely to cause buccal tilting of molars. However, the amount of tipping induced by RME is negligible, coming in at only 11.2 mm [23,32,33], which means that it can be ignored. The skeletal expansion produced by SME was not the same as that produced by RME [32]. In a manner analogous to that of the traditional hyrax expander, the RME that has differential apertures is able to augment the bone alterations. When there is a need for more considerable expansion in the front part of the maxillary arch, the differential expander is an adequate alternative to typical RME [34]. There have been reports that the Hyrax expander, an expander with deferential opening, and a fan-type expander are all capable of producing comparable skeletal modifications in children who have mixed dentition [35,36,37]. Researchers carried out trials with a variety of demographics and reported comparable findings in patients with clefts who used a variety of expanders [20,38,39,40,41,42,43,44,45,46].

According to a systematic review and meta-analysis [47] conducted on the topic, long-term results of the dentoalveolar effects show an increase in the transversal dimension with various levels of evidence for RME and SME, though there is insufficient evidence for skeletal changes using RME. RME and SME both induce the same amount of posterior expansion in cleft patients, but only SME promotes a significant amount of anterior differential expansion, according to the findings of another systematic analysis [47]. A recent systematic review reported that RME and SME produce the same amount of expansion in the posterior region when used in cleft patients. The review also opined that there is not sufficient evidence concerning the dental adverse effects of RME and SME in cleft patients [47]. This conclusion was reached as a result of the findings of the review. However, to obtain positive results in cleft patients and healthy persons, it is necessary to apply eruption guidance and expansion throughout the mixed dentition phase [48,49,50,51]. This is done to avoid dento-skeletal abnormalities. In the two studies that were available for evaluation, one study used study models [21] and the other relied on three-dimensional imaging [22]. In both investigations, the three-dimensional imaging study [22] was conducted using the same techniques, and the findings were analyzed both before the expansion and 4-6 months following it. In a separate piece of research [21], digital dental models that were obtained prior to the active growth period as well as six months following it were employed for the purpose of analysis. In BLCP patients with mixed dentition, the SMEs needed a longer complete maxillary expansion than the RMEs did. This was confirmed in both trials [21,22]. According to the findings of this paper, any option can be utilized by BLCP patients at the mixed dentition stage in order to increase the maxillary arch. The fact that there were only two published randomized controlled trials is the primary limitation of this review. The inclusion and exclusion criteria that were employed in the search were based on previous research. The fact that only patients with BCLP participated in the trial was another drawback of the investigation. According to the findings of prior research that evaluated various types of maxillary expanders, the Hyrax expander performed significantly better than the other RME types [45]. Only two studies were available in the literature, which shows the need for further studies. The appliances, such as W-arch and TAD-supported RME, need to be examined using well-developed procedures to evaluate how effectively they deliver the intended effects.

## 5. Conclusions

Both the RME and the SME are capable of producing comparable results in terms of maxillary expansion in patients with bilateral cleft palate who are at the mixed dentition stage; however, the SME requires more time than the RME. To adequately explain the specific roles of SME and RME in patients with bilateral cleft lip and palate, further research with a larger sample size is absolutely necessary.

## Figures and Tables

**Table 1:** The details of finally available studies.

Study	Type of Study	Participants	Intervention I	Intervention II	Observations
de Medeiros Alves et al., 2016 [21]	Parallel-arms RCT	50 (BCLP)	RME	SME	SME and RME increased the maxillary arch width. No statistically significant difference was found between the two techniques.
de Almeida et al., 2017 [22]	Parallel-arms RCT	46 (BCLP)	RME	SME	SME and RME produced an increase in maxillary arch width with no difference between the two techniques.

**Table 2:** The characteristics of the available studies.

Author	Age (years)	Gender		Method	Measurement 1	Measurement 2	Measurements
		SME	RME				
de Medeiros Alves et al. [21]	7 to 11	18 (M) 5 (F)	16 (M) 7(F)	Digital casts	Pre-expansion	6 months after active expansion	Arch perimeter, maxillary dental arch widths, arch length, the buccolingual inclination of posterior teeth palatal depth, and differential amount of expansion accomplished at the molar and canine regions.
de Almeida et al. [22]	8 to 10	19 (M) 6 (F)	18 (M) 7(F)	Cone beam computerized tomography images	Pre-expansion	4 to 6 months after the expansion	Maxillary width, nasal cavity width, arch width, alveolar crest width, palatal cleft width, an inclination of posterior teeth, buccal and lingual bone plate thickness, and alveolar crest level

M = males; F = females; RME = Rapid maxillary expansion; SME = Slow maxillary expansion.

## Data Availability

Not applicable.

## References

[1] Canfield M.A., Honein M.A., Yuskiv N., Xing J., Mai C.T., Collins J.S., Devine O., Petrini J., Ramadhani T.A., Hobbs C.A. (2006). National estimates and race/ethnic-specific variation of selected birth defects in the United States, 1999–2001. Birth Defects Res. Part A Clin. Mol. Teratol..

[2] Nagase Y., Natsume N., Kato T., Hayakawa T. (2010). Epidemiological Analysis of Cleft Lip and/or Palate by Cleft Pattern. J. Maxillofac. Oral Surg..

[3] Mossey P., Modell B. (2012). Epidemiology of Oral Clefts 2012: An International Perspective. Front. Oral Biol..

[4] IPDTOC Working Group, Jaruratanasirikul S., Chicharoen V., Chakranon M., Sriplung H., Limpitikul W., Dissaneevate P., Intharasangkanawin N., Tantichantakarun P., Sattapanyo A. (2011). Prevalence at Birth of Cleft Lip with or without Cleft Palate: Data from the International Perinatal Database of Typical Oral Clefts (IPDTOC). Cleft Palate-Craniofacial J..

[5] Dixon M.J., Marazita M.L., Beaty T.H., Murray J.C. (2011). Cleft lip and palate: Understanding genetic and environmental influences. Nat. Rev. Genet..

[6] Nasreddine G., El Hajj J., Ghassibe-Sabbagh M. (2021). Orofacial clefts embryology, classification, epidemiology, and genetics. Mutat. Res. Mol. Mech. Mutagen..

[7] Freitas J.A.D.S., Garib D.G., Oliveira M., Lauris R.D.C.M.C., de Almeida A.L.P.F., Neves L.T., Trindade-Suedam I.K., Yaedú R.Y.F., Soares S., Pinto J.H.N. (2012). Rehabilitative treatment of cleft lip and palate: Experience of the Hospital for Rehabilitation of Craniofacial Anomalies–USP (HRAC-USP)–Part 2: Pediatric Dentistry and Orthodontics. J. Appl. Oral Sci..

[8] Park Y.J., Derderian C., Oppedisano M. (2021). Interdisciplinary Approach for the Treatment of Complex Bilateral Cleft Lip and Palate With Missing Premaxilla. Cleft Palate-Craniofacial J..

[9] Semb G. (1991). A study of facial growth in patients with bilateral cleft lip and palate treated by the Oslo CLP Team. Cleft Palate-Craniofacial J. Off. Publ. Am. Cleft Palate-Craniofacial Assoc..

[10] Filho L.C., De Almeida A.M., Ursi W.J. (1994). Rapid maxillary expansion in cleft lip and palate patients. J. Clin. Orthod. JCO.

[11] Sakamoto T., Sueishi K., Miyazaki H., Katada H., Ebihara T., Kosaka T. (2008). Clinical statistical investigation of cleft lip and palate patients aged over 18 years at Department of Orthodontics, Suidobashi Hospital, Tokyo Dental College. Bull. Tokyo Dent. Coll..

[12] Baek S.H., Moon H.S., Yang W.S. (2002). Cleft type and Angle’s classification of malocclusion in Korean cleft patients. Eur. J. Orthod..

[13] Vettore M.V., Campos A.E.S. (2010). Malocclusion characteristics of patients with cleft lip and/or palate. Eur. J. Orthod..

[14] Torres A., AlYazeedy I., Yen S. (2019). A Programmable Expander for Patients With Cleft Lip and Palate. Cleft Palate-Craniofacial J..

[15] de Oliveira Cavassan A., de Albuquerque M.D., Filho L.C.F. (2004). Rapid maxillary expansion after secondary alveolar bone graft in a patient with bilateral cleft lip and palate. Cleft Palate-Craniofacial J..

[16] Simsuchin C., Chen Y., Mallineni S.K. (2023). Clinical Effectiveness of Vestibular Shields in Orthodontic Treatment: A Scoping Review. Children.

[17] Huang L., Huang N., Deng X., Chen X. (2012). [Semi-attached quad-helix appliance can expand the maxillary arches in patients with cleft lip and palate before alveolar bone grafting]. Hua Xi Kou Qiang Yi Xue Za Zhi.

[18] Petrén S., Bondemark L., Söderfeldt B. (2003). A systematic review concerning early orthodontic treatment of unilateral posterior crossbite. Angle Orthod..

[19] Caroccia F., Moscagiuri F., Falconio L., Festa F., D’attilio M. (2020). Early Orthodontic Treatments of Unilateral Posterior Crossbite: A Systematic Review. J. Clin. Med..

[20] Vizzotto M.B., de Araújo F.B., da Silveira H.E.D., Boza A.A., Closs L.Q. (2008). The Quad-Helix Appliance in the Primary Dentition –Orthodontic and Orthopedic Measurements. J. Clin. Pediatr. Dent..

[21] Alves A.C.D.M., Garib D.G., Janson G., de Almeida A.M., Calil L.R. (2016). Analysis of the dentoalveolar effects of slow and rapid maxillary expansion in complete bilateral cleft lip and palate patients: A randomized clinical trial. Clin. Oral Investig..

[22] de Almeida A.M., Ozawa T.O., de Medeiros Alves A.C., Janson G., Lauris J.R.P., Ioshida M.S.Y., Garib D.G. (2017). Slow versus rapid maxillary expansion in bilateral cleft lip and palate: A CBCT randomized clinical trial. Clin. Oral Investig..

[23] Brunetto M., Andriani J.D.S.P., Ribeiro G.L.U., Locks A., Correa M., Correa L.R. (2013). Three-dimensional assessment of buccal alveolar bone after rapid and slow maxillary expansion: A clinical trial study. Am. J. Orthod. Dentofac. Orthop..

[24] Cassi D., Di Blasio A., Gandolfinini M., Magnifico M., Pellegrino F., Piancino M.G. (2017). Dentoalveolar effects of early or-thodontic treatment in patients with cleft lip and palate. J. Craniofacial Surg..

[25] Vasant M.R., Menon S., Kannan S. (2009). Maxillary Expansion in Cleft Lip and Palate using Quad Helix and Rapid Palatal Expansion Screw. Med. J. Armed Forces India.

[26] Ayub P.V., Garib D.G., Ebrahim H., Polido J., Blasca W., Yen S. (2022). Intercenter comparison of slow and rapid maxillary expansion in unilateral complete cleft lip and palate. Dent. Press J. Orthod..

[27] Lagravère M.O., Ling C.P., Woo J., Harzer W., Major P.W., Carey J.P. (2020). Transverse, vertical, and anterior-posterior changes between tooth-anchored versus Dresden bone-anchored rapid maxillary expansion 6 months post-expansion: A CBCT randomized controlled clinical trial. Int. Orthod..

[28] Garib D.G., Henriques J.F.C., Janson G., Freitas M.R., Coelho R.A. (2005). Rapid maxillary expansion–tooth tissue-borne versus tooth-borne expanders: A computed tomography evaluation of dentoskeletal effects. Angle Orthod..

[29] Gregório L., Alves A.C.d.M., de Almeida A.M., Naveda R., Janson G., Garib D. (2019). Cephalometric evaluation of rapid and slow maxillary expansion in patients with BCLP: Secondary data analysis from a randomized clinical trial. Angle Orthod..

[30] Bastos R.T.D.R.M., Blagitz M.N., Aragón M.L.S.D.C., Maia L.C., Normando D. (2019). Periodontal side effects of rapid and slow maxillary expansion: A systematic review. Angle Orthod..

[31] Pereira J.D.S., Jacob H.B., Locks A., Brunetto M., Ribeiro G.L.U. (2017). Evaluation of the rapid and slow maxillary expansion using cone-beam computed tomography: A ran-domized clinical trial. Dental Press J. Orthod..

[32] Greenbaum K.R., Zachrisson B.U. (1982). The effect of palatal expansion therapy on the periodontal supporting tissues. Am. J. Orthod..

[33] Martina R., Cioffi I., Farella M., Leone P., Manzo P., Matarese G., Portelli M., Nucera R., Cordasco G. (2012). Transverse changes determined by rapid and slow maxillary expansion–A low-dose CT-based randomized controlled trial. Orthod. Craniofac. Res..

[34] Garib D., Lauris R.D.C.M.C., Calil L.R., Alves A.C.D.M., Janson G., De Almeida A.M., Cevidanes L.H.S., Lauris J.R.P. (2016). Dentoskeletal outcomes of a rapid maxillary expander with differential opening in patients with bilateral cleft lip and palate: A prospective clinical trial. Am. J. Orthod. Dentofac. Orthop..

[35] Alves A.C.D.M., Janson G., Mcnamara J.A., Lauris J.R.P., Garib D.G. (2020). Maxillary expander with differential opening vs Hyrax expander: A randomized clinical trial. Am. J. Orthod. Dentofac. Orthop..

[36] Goje S.K. (2020). Expander with differential opening. Am. J. Orthod. Dentofac. Orthop..

[37] Massaro C., Garib D., Cevidanes L., Janson G., Yatabe M., Lauris J.R.P., Ruellas A.C. (2021). Maxillary dentoskeletal outcomes of the expander with differential opening and the fan-type expander: A randomized controlled trial. Clin. Oral Investig..

[38] Yang C.-J., Pan X.-G., Qian Y.-F., Wang G.-M. (2012). Impact of rapid maxillary expansion in unilateral cleft lip and palate patients after secondary alveolar bone grafting: Review and case report. Oral Surg. Oral Med. Oral Pathol. Oral Radiol..

[39] Lilja J., Kalaaji A., Friede H., Elander A. (2000). Combined Bone Grafting and Delayed Closure of the Hard Palate in Patients with Unilateral Cleft Lip and Palate: Facilitation of Lateral Incisor Eruption and Evaluation of Indicators for Timing of the Procedure. Cleft Palate Craniofacial J..

[40] Fastuca R., Michelotti A., Nucera R., D’Antò V., Militi A., Logiudice A., Caprioglio A., Portelli M. (2020). Midpalatal Suture Density Evaluation after Rapid and Slow Maxillary Expansion with a Low-Dose CT Protocol: A Retrospective Study. Medicina.

[41] Costa J.G., Galindo T.M., Mattos C.T., Cury-Saramago A.A. (2017). Retention period after treatment of posterior crossbite with maxillary expansion: A systematic review. Dent. Press J. Orthod..

[42] Singh H., Maurya R.K., Sharma P., Kapoor P., Mittal T., Atri M. (2021). Effects of maxillary expansion on hearing and voice function in non-cleft lip palate and cleft lip palate patients with transverse maxillary deficiency: A multicentric randomized controlled trial. Braz. J. Otorhinolaryngol..

[43] Pugliese F., Palomo J.M., Calil L.R., de Medeiros Alves A., Lauris JR P., Garib D. (2020). Dental arch size and shape after maxillary expansion in bilateral complete cleft palate: A comparison of three expander designs. Angle Orthod..

[44] Shahab N., Sar C., Sarac M., Erverdi N. (2019). Reconstruction of Premaxilla With Alveolar Distraction Osteogenesis in a Patient With Complete Cleft Lip and Palate: A Case Report. Cleft Palate-Craniofacial J. Off. Publ. Am. Cleft Palate-Craniofacial Assoc..

[45] Figueiredo D.S.F., Bartolomeo FU C., Romualdo C.R., Palomo J.M., Horta MC R., Andrade I., Oliveira D.D. (2014). Dentoskeletal effects of 3 maxillary expanders in patients with clefts: A cone-beam computed tomography study. Am. J. Orthod. Dentofac. Orthop..

[46] Lsawaf D.H., Almaasarani S.G., Hajeer M.Y., Rajeh N. (2022). The effectiveness of the early orthodontic correction of functional unilateral posterior crossbite in the mixed dentition period: A systematic review and meta-analysis. Prog. Orthod..

[47] Luyten J., De Roo NM C., Christiaens J., Van Overberghe L., Temmerman L., De Pauw G.A. (2023). Rapid maxillary expansion vs slow maxillary expansion in patients with cleft lip and/or palate: A systematic review and meta-analysis. Angle Orthod..

[48] Huang S., Chen Y., Chen T., Mallineni S.K., McGrath C., Hagg U. (2022). Clinical effectiveness of the Eruption Guidance Appliances in treating malocclusion in the mixed dentition: A systematic review and meta-analysis. Int. J. Paediatr. Dent..

[49] Alam M.K., Alfawzan A.A. (2020). Dental Characteristics of Different Types of Cleft and Non-cleft Individuals. Front. Cell Dev. Biol..

[50] Bucci R., D’Antò V., Rongo R., Valletta R., Martina R., Michelotti A. (2016). Dental and skeletal effects of palatal expansion techniques: A systematic review of the current evidence from systematic reviews and meta-analyses. J. Oral Rehabil..

[51] Kapila S.D., Nervina J.M. (2015). CBCT in orthodontics: Assessment of treatment outcomes and indications for its use. Dento Maxillo Facial Radiol..

